# Supplementary material for: Blockade of Wnt Secretion Attenuates Myocardial Ischemia–Reperfusion Injury by Modulating the Inflammatory Response
Source: Int J Mol Sci. 2022 Oct 14;23(20):12252. doi: 10.3390/ijms232012252 (PMC9602582; doi:10.3390/ijms232012252)
Supplement: Supplementary file 1 [file ijms-23-12252-s001.zip › ijms-1919709-supplementary.pdf]

S1

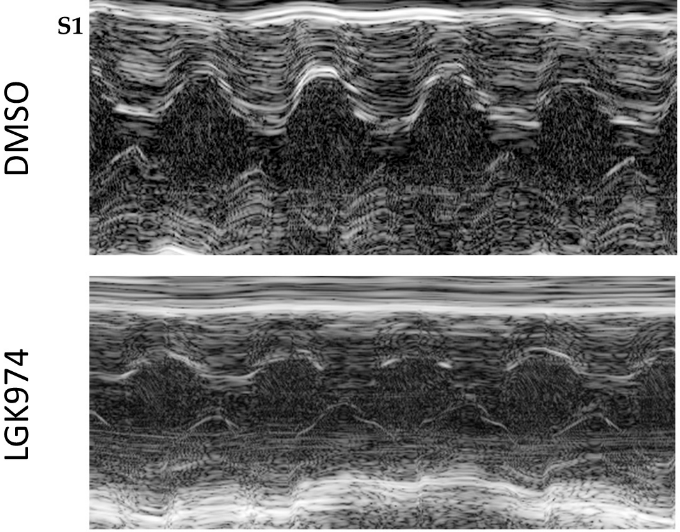
DMSO
LGK974

S2

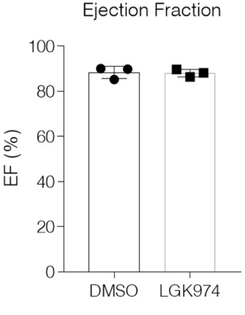
Ejection Fraction
EF (%)
100
80
60
40
20
0
DMSO
LGK974

S3

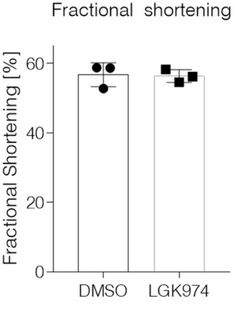
Fractional shortening
Fractional Shortening [%]
60
40
20
0
DMSO
LGK974

S4

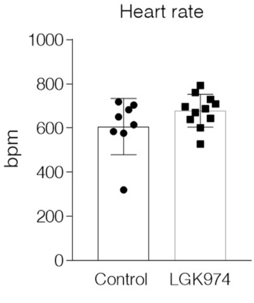
Heart rate
bpm
1000
800
600
400
200
0
Control
LGK974

S5

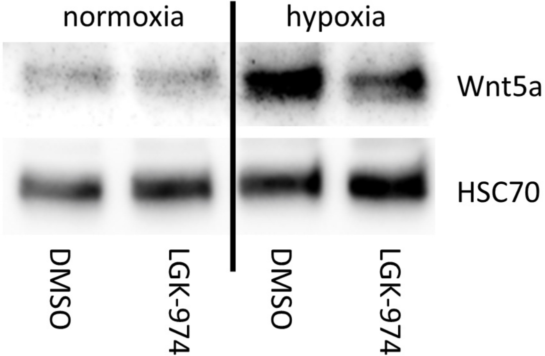

S6

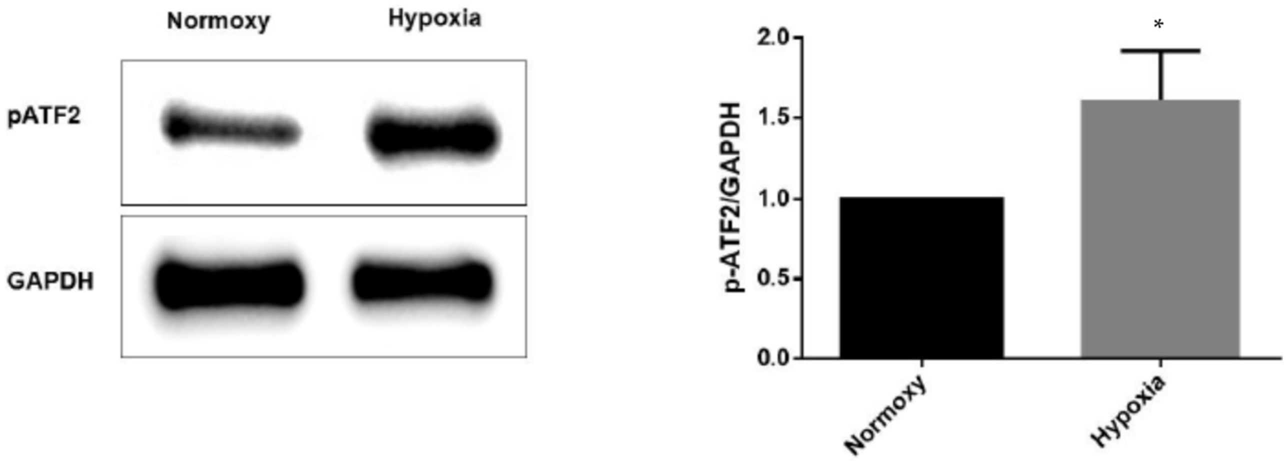

Normoxy
Hypoxia
pATF2
GAPDH
p-ATF2/GAPDH
2.0
1.5
1.0
0.5
0.0
*
Normoxy
Hypoxia

Supplemental figure S 1-4: S1: Representative M-Mode echocardiographic images of DMSO (top) and LGK-974 treated (bottom) animals under basal conditions. S2 and S3: Echocardiographic results from DMSO and LGK-974 treated animals under basal conditions. S4: Heart rate of LGK-974 and DMSO treated animals 28 days after I/R injury.

Supplemental figure S5: Western blot of Wnt5A from supernatant of cardiomyocytes cultured under hypoxic or normoxic condition in the presence or absence of LGK-974. HSC70 were used as a secreted control protein. LGK-974 effectively inhibited Wnt5A secretion.

Supplemental figure S 6: Stimulation of RAW264.7 cell with supernatant of hypoxic cardiomyocytes leads to activation of non-canonical Wnt signaling as seen by increased pATF2 expression levels.
